# Phytolacca Decandra in Mastitis

**Published:** 1874-06

**Authors:** T. Curtis Smith


PHYTOLACCA DECANDRA IN MASTITIS—*By T. Curtis Smith, M.D.* * * * She was again confined in December, 1873, and again was likely to be beset with a series of abscesses as before. After several days of fruitless efforts to abate the threatening suppurative process, I placed her on fifteen-drop doses of a saturated tincture of the root of phytolacca decandra, every four hours, and applied the same freely over the glands. All other treatment for this purpose being withdrawn. Contrary to my expectations, the inflammation began to abate in twenty-four hours, and in three days the glands were soft throughout, and the lacteal secretion completely dried up. The latter point I did not expect to attain with this agent, but, as the milk was not healthy for the child, it was better so than otherwise. She is still very feeble, anæmic, and a great sufferer from a most obstinate neuralgia. It will be readily seen that everything was favorable for the development of mastitis, which I have learned had occurred after several of her previous confinements.

